# Prevalence of Hypertension in the Middle Belt of Ghana: A Community-Based Screening Study

**DOI:** 10.1155/2019/1089578

**Published:** 2019-10-07

**Authors:** David Kwame Dosoo, Solomon Nyame, Yeetey Enuameh, Harold Ayetey, Harry Danwonno, Mieks Twumasi, Cephas Tabiri, Stephaney Gyaase, Gregory Y. H. Lip, Seth Owusu-Agyei, Kwaku Poku Asante

**Affiliations:** ^1^Kintampo Health Research Centre, Ghana Health Service, Kintampo, Ghana; ^2^Kwame Nkrumah University of Science and Technology, Kumasi, Ghana; ^3^University of Cape Coast School of Medical Sciences, Cape Coast Ghana, Ghana; ^4^Royal Brompton and Harefield NHS Trust, London, UK; ^5^Liverpool Centre for Cardiovascular Science, University of Liverpool and Liverpool Heart & Chest Hospital, Liverpool, UK; ^6^Aalborg Thrombosis Research Unit, Department of Clinical Medicine, Aalborg University, Aalborg, Denmark; ^7^Institute of Health Research, University of Health and Allied Sciences, Ho, Ghana

## Abstract

**Objective:**

Prevalence of hypertension is on the rise and can be attributed to aging populations and changing behavioral or lifestyle risk factors. The objectives of this study were to determine the prevalence, awareness, treatment, control, and risk factors of hypertension in the middle part of Ghana.

**Methods:**

A total of 2,555 participants aged ≥18 years (mean age of 43 years; 60.5% female) were enrolled using a two-stage sampling method. The World Health Organization STEPwise approach to chronic disease risk factor Surveillance-Instrument v2.1 was used for data collection. Blood pressure and anthropometric measurements were assessed. Blood glucose and lipids were also measured using blood samples collected after an overnight fast.

**Results:**

Prevalence of hypertension was 28.1% (95% CI: 26.3%–29.8%). Less than half, i.e., 45.9% (95% CI: 42.2%–49.6%), of the respondents were aware of their hypertensive status. Of those aware and had sought medical treatment, 41.3% (95% CI: 36.1–46.8) had their hypertension controlled. Risk factors associated with being hypertensive were current (*p*=0.053) and past tobacco usage (*p* < 0.001), prediabetes (*p*=0.042), high body mass index (*p* < 0.001), hyperglycaemia (*p*=0.083), and hypercholesterolaemia (*p*=0.010). Doing vigorous work and being active in sports were less associated with being hypertensive (*p* < 0.001).

**Conclusion:**

Our study showed that close to one-quarter of adults who were involved in the survey in the middle belt of Ghana were hypertensive with less than half being aware of their hypertensive status; nearly half of those on treatment had controlled hypertension. Healthcare systems need adequate resources that enable them to screen, educate, and refer identified hypertensive patients for appropriate management to prevent or minimize the development of hypertension-related complications.

## 1. Introduction

Hypertension is a major public health challenge, affecting populations from both economically developed and developing countries globally [1, 2]. The disease remains a major risk factor for development of cardiovascular disease (CVD), which was responsible for 17.7 million deaths worldwide in 2015 (i.e., 45% of deaths due to noncommunicable diseases) [3]. The highest prevalence of hypertension in the world occurs in sub-Saharan Africa [4]. Many patients with hypertension are asymptomatic and predisposed to fatal cardiovascular events such as strokes and myocardial infarctions; hence, the the disease is described as a “silent killer” [5]. Depending on the aetiology, hypertension could be classified as primary (affecting 90–95% of patients) or secondary (resulting from an underlying condition) [5].

Although the exact cause of primary hypertension is unknown, there are several nonmodifiable and modifiable risk factors that have been linked to the condition. Nonmodifiable risk factors for hypertension include age, sex, race, family history, and genetic composition, whereas modifiable risk factors include obesity, excessive salt intake, inactivity or lack of exercise, high fat diet, tobacco use, and alcohol consumption [5–7]. According to the 2014 Ghana Demographic and Health Survey (GDHS), the overall prevalence of hypertension among persons aged 15–49 years was 13.0%, with nearly half of them being aware of their hypertensive status [8]. A systematic review and meta-analysis by Bosu also reported a prevalence of adult hypertension between 19% and 48% [9].

Although the prevalence of hypertension was determined as part of the GDHS in 2014, this survey did not include biochemical measurements as outlined in the WHO STEPwise approach to chronic disease risk factor Surveillance-Instrument v2 due in part to cost considerations. A well-structured epidemiological survey to determine the prevalence, management, and risk factors including biochemical risk are vital to compliment that of the GDHS for the implementation and evaluation of the impact of interventions to control hypertension. A multiphased Kintampo Noncommunicable diseases (NCD) Initiative (KNI) programme incorporating the WHO STEPwise approach was established at the Kintampo Health Research Centre (KHRC) in 2015 to build capacity for NCD epidemiologic studies that would lead to interventional studies. This community-based epidemiological screening survey that establishes the burden of hypertension in the middle belt of Ghana initiated the programme.

## 2. Methods

### 2.1. Study Design and Setting

A community-based cross-sectional study was conducted in two contiguous districts (Kintampo North Municipality and Kintampo South District) in the middle part of Ghana between October 2015 and December 2016. The study area, which lies in the geographical center of Ghana, has a total resident population of about 129,000 of which about two-thirds is rural [10]. There were two government hospitals, two private hospitals, four health centers, one private clinic, 25 community-based health planning and services (CHPS) areas, and two maternity homes that serve the population. The KHRC maintains a Health and Demographic Surveillance System (HDSS), which collects core information such as births, deaths, and migrations (in and out). Houses within the study area have been numbered and digitized, facilitating tracing of potential study participants in their homes [10].

Five days of training was organized for the clinical, laboratory, field, and data teams involved in the study covering implementation of the study protocol, specifically interviewing techniques, informed consenting process, anthropometric measurements, completion of the study questionnaire, and referral systems. Theoretical presentations and role play were employed during training sessions. Study tools were piloted within the community prior to commencement of the study.

### 2.2. Ethical Considerations

Ethical approval was obtained from the Kintampo Health Research Centre Institutional Ethics Committee (KHRCIEC/2014–25). Individual written informed consent was obtained from each participant before study procedures were carried out.

### 2.3. Sample Size

Based on an overall hypertension prevalence of 13.0% in the Ghanaian population status [8], a prevalence of 9.5% was used for the study area. Assuming a 0.05 type I error rate and a 0.011 margin of error, a minimum sample of 2,482 was estimated. Assuming a 10% nonresponse rate, a sample size of 2,730 was used. The sample size calculation was based on the following formula:

(1) $$\begin{array}{c} n=Z_{1-\left(\alpha /2\right)}^{2}\frac{P\left(1-P\right)}{d^{2}}, \end{array}$$

where *n* = sample size, *Z*_1−(*α*/2)_ = type I error rate (0.05 = 1.96), *P* = assumed hypertension prevalence (13.0%), and *d* = margin of error (0.011).

### 2.4. Selection of Study Communities and Participants

The sampling frame was the database of the Kintmapo Health and Demographic Surveillance System (KHDSS). A two-stage sampling procedure was used in selecting the study population, ensuring that they were representative of the KHDSS population. In the first stage, 34 of the 164 communities in the KHDSS area were randomly selected using computer-generated random numbers and ensuring that the entire target population was covered. This was followed by visits to the leaders of the selected communities to explain the study to them and to seek their permission for participation of members' of their communities. The second stage involved stratification by gender and ascending age, of all individuals of 18 years and older in the selected communities. Potential study participants were then selected at random from the identified communities using a systematic sampling technique, which involved the selection of an “indexed” individual from the database of each selected community followed by the selection of every tenth individual. Listings of potential study participants were used by our study field workers who met the participants at home in the communities to explain the study to them and also invite them to a central location within the community for them to go through study procedures. Inclusion criteria were adult male and female subjects aged ≥18 years who have been resident in the study area for at least 6 months and were willing to be part of the study.

### 2.5. Study Procedures

Following informed consent, questionnaires were administered to obtain demographic data, information on tobacco use, alcohol consumption, diet, physical activity, history of raised blood pressure, and diabetes, using the World Health Organization (WHO) STEPwise approach to chronic disease risk factor Surveillance-Instrument v2.1 [11]. All administered questionnaires were checked for completeness in the field and before data entry.

### 2.6. Blood Pressure Measurement

Three sedentary blood pressure (BP) measurements were taken at 3-minute intervals by a physician assistant using an Omron digital BP monitor (Omron Healthcare Co. Kyoto, Japan) with a cuff of appropriate width after the participant had sat for at least 15 minutes and relaxed.

### 2.7. Body Weight and Height Measurement

Two trained field staff measured and verified the body weight and height with the participant standing motionless in an erect position against the vertical surface of a standard height meter attached to a calibrated weighing scale. Participants had on minimum clothing and no footwear while their body weight and height were being measured.

### 2.8. Laboratory Measurements

Overnight fasting blood samples (3 ml) were obtained by sterile venipuncture from each participant and dispensed into 1 ml fluoride-EDTA and 2 ml lithium heparin tubes for the estimation of glucose and lipids, respectively. Plasma was separated after centrifugation of blood at 1000*g* for 10 min. Glucose, cholesterol, and triglyceride levels were determined the same day using a Flexor *E* automated Clinical Chemistry analyzer (Vital Scientific, The Netherlands) according to approved standard operating procedures. Samples that were to be analyzed later were kept frozen at −20°C or below and thawed prior to analysis. Freeze-thaw cycles were avoided.

### 2.9. Outcome Definitions

We used definitions by the Seventh Report of the Joint National Committee on the Prevention, Detection, Evaluation and Treatment of High Blood Pressure [12]. Hypertension was defined as a mean systolic blood pressure (SBP) of ≥140 mmHg and/or a mean DBP of ≥90 mmHg or a respondent self-report of clinician diagnosis of hypertension or self-report of medication to lower blood pressure. Prehypertension was also defined as SBP of 120–139 mmHg and DBP of 80–89 mmHg; stage 1 hypertension as SBP of 140–159 mmHg and/or DBP 90–99 mmHg; and stage 2 hypertension as SBP of ≥160 mmHg and/or DBP ≥100 mmHg. Awareness was defined as self-report of a diagnosis of hypertension by a health worker. Control was defined as self-reported taking of medication to lower blood pressure with SBP of <140 mmHg and DBP of <90 mmHg. Aware yet not on medication was defined as self-reported diagnosis by a health worker yet not taking medication to lower blood pressure. Diabetes mellitus was defined as fasting blood glucose level of ≥7.0 mmol/L or reported use of blood glucose lowering medication, whereas hypercholesterolemia and hypertriglyceridemia were defined as total cholesterol and triglyceride levels of ≥5.0 mmol/L and ≥2.2 mmol/L, respectively.

Participants found to be hypertensive or having any other medical condition were counseled by a study clinician and referred to a health facility for treatment. All participants with a measured SBP of ≥140 mmHg and/or DBP of ≥90 mmHg were referred to the Municipal/District hospital or to their clinicians for appropriate management.

### 2.10. Data Management, Statistical Methods, and Analysis

Data were double entered, verified using (C Sharp) Visual Studio 2013 as front end and SQL server as back end prior to analysis. Means of second and third blood pressure measurements were used for the analysis. Measurement of household socioeconomic status was based on respondent's household assets. The assets were scored, and eigenvalues were computed using principal component analysis. By this method, the weighted assets were grouped into various components. The first component was used to generate the wealth quintiles. Variables were summarized using descriptive statistics. Chi-square test was used to find the relationship between being hypertensive and known risk factors. The analysis was done in Stata version 14.0 (StataCorp, USA).

## 3. Results

A total of 2,555 participants (60.5% female) from the HDSS area were enrolled into the survey following consenting (Table 1). Participants' enrolment was proportional to the population structure. For the risk set, those aged 18–39 years were in the majority (45.3%), followed by those aged 40–59 years (35.7%). Approximately 41% of participants had no education, and 15.6% had a postsecondary educational status (e.g., training colleges and polytechnics) as their highest educational level. Majority (29%) of the participants were from households with least poor socioeconomic status.

### 3.1. Prevalence of Hypertension

The overall prevalence of hypertension in the surveyed population was 28.1% (95% CI: 26.3–29.8%; 717/2555), and this increased with age. Age-specific prevalence of hypertension for participants aged 18–39 years, 40–59 years, and ≥60 years were 12.9% (95% CI: 11.1–15.0%), 33.8% (95% CI: 30.8–37.0%), and 53.3% (95% CI: 48.8–57.7%), respectively.

Prevalence of the different types of raised blood pressure, i.e., prehypertension, systolic hypertension, diastolic hypertension, stage 1 hypertension, and stage 2 hypertension were 42.5% (95% CI: 40.6%–44.5%), 8.5% (95% CI: 7.5%–9.7%), 3.2% (95% CI: 2.6%–3.9%), 15.8% (95% CI: 14.4%–17.2%), and 8.9% (95% CI: 7.9%–10.1%), respectively (Table 2).

Diastolic hypertension was the least common type of hypertension in the population but was most prevalent within 40–59 years. With the exception of diastolic hypertension, participants aged ≥60 years generally had the most prevalent proportion for all the various types of hypertension. Close to a fifth (19%) of those aged ≥60 years had stage 2 hypertension (Table 2).

Prevalence of the different stages of hypertension in male and female subjects is presented in Table 3. Male subjects had a significantly higher prevalence of prehypertension (*p* < 0.001) and stage-1 hypertension (*p*=0.002) compared with female subjects.

### 3.2. Awareness, Treatment, and Control of Hypertension

Out of the 717 respondents who were classified as hypertensive, 45.9% (95% CI: 42.2–49.6; 329/717) had in the past been diagnosed and informed by a health practitioner and were aware of their status (Table 4). Level of awareness increased significantly with age group (*p* < 0.001), being highest in the ≥60 years group and lowest in the 18–39 years group. Out of the 329 hypertensive respondents who were aware of their status, 54.1%, 3.6%, and 2.4% of them had sought for medical, herbal, and both medical and herbal treatments, respectively. Among those who sought medical treatment, 41% had their raised blood pressure controlled, while 59% had uncontrolled blood pressure. A higher proportion of participants aged ≥60 years had sought treatment (Table 4).

### 3.3. Risk Factors for Hypertension

Current usage of tobacco was highest (3.2%) among the age group of 40–59 years' compared with the other age categories ([Supplementary-material supplementary-material-1]). Hypertensive respondents who have ever or within the past 12 months or within the past 30 days consumed alcohol were approximately 38%, 22%, and 19%, respectively. Consumption of alcohol within the past 30 days was approximately 18% for each of the age groups ([Supplementary-material supplementary-material-1]).

Approximately 85% and 21% of hypertensive respondents were involved in active work and sports, respectively. Approximately 10% of these respondents also had diabetes, with the prevalence being highest (11.0%) for respondents within the 40–59 years group. About half of the hypertensive respondents had a normal BMI. Majority of hypertensive respondents had hypercholesterolemia, with proportions being highest (59.9%) for respondents of ≥60 years. Proteinuria (≥0.3 g/L) was present in 3.5% (25/714) of hypertensive patients ([Supplementary-material supplementary-material-1]).

Risk factors found to be associated with one being hypertensive were current tobacco usage (*p*=0.053), past tobacco usage (*p* < 0.001), prediabetes (*p*=0.042), high BMI (*p* < 0.001), hyperglycaemia (*p*=0.038), and hypercholesterolaemia (0.010), whereas being active in sports and doing vigorous work were both less associated with hypertension (*p* < 0.001) ([Supplementary-material supplementary-material-1]). The risk factors were the same for both male and female subjects across the different age groups.

## 4. Discussion

This community-based epidemiological screening study establishes that the prevalence of hypertension is higher than expected in predominantly rural communities in Ghana. The overall prevalence was consistent with what was reported by Bosu as the prevalence of hypertension among adults in Ghana [9], Mohammed et al. among Kenyans [7], and Arrey et al. among Cameroonians [13]. Our value, however, was lower compared with those from other cross-sectional studies in Cameroon [14, 15], Ghana [16], Kenya [17], South Africa [16, 18], and Tanzania [19], where the overall prevalence of hypertension was more than 40% of the study populations. The high prevalence of hypertension in this rural setting could be attributed to the changing lifestyles of rural residents to increasingly reflect those of urban dwellers, including alcohol abuse, smoking, lack of physical exercise, and high intake of salt and fatty foods. The increased prevalence of hypertension with age reported in this study has been well documented in other studies [1, 20–22]. Arterial stiffening as a result of aging has been described as a contributory factor for the higher predisposition to hypertension among the elderly [23–25].

Our demonstration that more than half of participants with hypertension were not aware of their condition is in agreement with findings in several other studies [4, 7, 26]. Low access to healthcare services and poor health seeking behavior could be contributing factors to the low levels of awareness. The high proportion of persons identified with hypertension and not on treatment reported in our study is similar to the 59.5% reported by Sanuade et al. [8] in a national survey in Ghana but lower compared with the 84.4% reported by Mohammed et al. [7] from a national survey in Kenya.

Our findings on the proportion of controlled hypertension in the different age groups were similar to the United States National Health and Nutrition Examination Surveys (NHANES) 1999–2014, which also showed lowest level of control among persons 18–39 years [21]. The nearly hypertension control rate reported in our study was similar to the 51% reported by Mohamed et al. in Kenya [7] but higher than the nearly one-third prevalence of controlled hypertension in the study by Khader et al. in Jordan [27] and the 23.8% reported by Sanuade et al. in a national survey in Ghana [8]. Although not optimal, the higher level of hypertension control compared with others could be due to higher levels of compliance to treatment regimens by persons with hypertension in our study area.

The high proportion of persons who knew they were hypertensive but were not on any treatment could be due to poor adherence and subsequent default to treatment. This has been attributed to factors such as inadequate counseling, misconceptions that hypertension is curable, and high cost of drugs [28–30]. More education on hypertension prevention and treatment through task shifting at the community level, taking hypertension (and other NCDs) healthcare to the doorsteps of community members, financing, and availability of hypertension medication through the National Health Insurance Scheme (NHIS) will contribute significantly to improvements in treatment rates among hypertensive individuals who are aware of their status [31]. Factors associated with hypertension in our study area were similar to findings from other studies [32, 33]. Health education and other interventions should target these risk factors to improve hypertension prevention and management.

## 5. Limitations

A key limitation is that some responses were subjective and self-reported. These responses could have been affected by recall bias. However, the use of well-trained interviewing staff and Show Cards to explain the questions ensured that, as much as possible, accurate responses were obtained.

## 6. Conclusions

Our study showed that close to one-quarter of adults in the middle belt of Ghana had hypertension with less than half of them being aware of their hypertensive status, and nearly half of those on treatment having controlled hypertension. Healthcare systems need adequate resources that will enable them to screen, educate, and refer identified hypertensive individuals for appropriate management to prevent or minimize the development of complications.

## Figures and Tables

**Table 1:** Distribution of respondents' basic demographic characteristics.

Demographic characteristics	Respondent's age group			Overall total (2,555)
	18–39 years	40–59 years	60 and above years	*n* (%)
	*N* = 1,158	*N* = 913	*N* = 484	
	*n* (%)	*n* (%)	*n* (%)	
Gender				
Male	441 (38.1)	353 (38.7)	215 (44.4)	1,009 (39.5)
Female	717 (61.9)	560 (61.3)	269 (55.6)	1,546 (60.5)
Residence type				
Urban	467 (40.3)	293 (32.1)	151 (31.2)	911 (35.7)
Rural	691 (59.7)	620 (67.9)	333 (68.8)	1,644 (64.3)
Education status				
None	246 (21.2)	455 (49.8)	342 (70.7)	1,043 (40.8)
Primary	194 (16.7)	131 (14.3)	42 (8.7)	367 (14.4)
Secondary	436 (37.6)	238 (26.1)	73 (15.1)	747 (29.2)
Postsecondary	282 (24.3)	89 (9.7)	27 (5.6)	398 (15.6)
Registered with NHIS^1^				
Yes	484 (41.8)	464 (50.8)	290 (59.9)	1,238 (48.4)
No	674 (58.2)	449 (49.2)	194 (40.1)	1,317 (51.6)
HH SES^2^				
Least poor	330 (28.5)	272 (29.8)	144 (29.7)	746 (29.2)
Less poor	287 (24.8)	215 (23.5)	122 (25.2)	624 (24.4)
Poor	229 (19.8)	147 (16.1)	86 (17.8)	462 (18.1)
More poor	155 (13.4)	154 (16.9)	64 (13.2)	373 (14.6)
Most poor	140 (12.1)	108 (11.8)	65 (13.4)	313 (12.2)
Missing	17 (1.5)	17 (1.9)	3 (0.6)	37 (1.4)

^1^NHIS: National Health Insurance Scheme; ^2^HH SES: household socioeconomic status.

**Table 2:** Various types of hypertension prevalence by age group within the surveyed population.

Raised blood pressure type	Respondent's age group			Total (2,555)
	18–39 years (*N* = 1,158)	40–59 years (*N* = 913)	60 and above years (*N* = 484)	
	% (95% CI)	% (95% CI)	% (95% CI)	% (95% CI)
Prehypertension	38.6 (35.8–41.1)	44.5 (41.3–47.7)	48.3 (43.9–52.8)	42.5 (40.6–44.5)
Systolic hypertension	3.4 (2.5–4.6)	8.2 (6.6–10.2)	21.5 (18.0–25.4)	8.5 (7.5–9.7)
Diastolic hypertension	3.0 (2.2–4.2)	3.9 (2.8–5.4)	2.3 (1.3–4.1)	3.2 (2.6–4.0)
Hypertension (stage 1)	8.3 (6.8–10.0)	18.8 (16.4–21.5)	27.9 (24.1–32.1)	15.8 (14.4–17.2)
Hypertension (stage 2)	2.3 (1.6–3.4)	12.0 (10.1–14.3)	19.0 (15.7–22.7)	9.0 (7.9–10.1)

**Table 3:** Prevalence of the different stages of hypertension among male and female subjects in the study area.

Stage of hypertension	Male	Female	Difference	*P* value
Prehypertension	49.3	38.1	11.3	<0.001
Systolic hypertension	9.3	8.0	1.3	0.252
Diastolic hypertension	3.3	3.2	0.1	0.887
Stage 1 hypertension	18.5	14.0	4.6	0.002
Stage 2 hypertension	8.9	9.0	−0.1	0.951

**Table 4:** Awareness, treatment, and control of hypertension among the study population.

	HPT (*N*)	Aware	Aware, % (95% CI)	Treated and controlled, % (95% CI)	Aware, not treated % (95% CI)
Age group (years)					
18–39	150	54	36.0 (28.7–44.0)	18.5 (10.2–31.3)	81.4 (68.7–89.8)
40–59	309	148	47.9 (42.4–53.5)	37.2 (29.7–45.3)	62.8 (54.7–70.3)
≥60	258	127	49.2 (43.1–55.3)	55.9 (47.1–64.3)	44.1 (35.6–52.9)
All	717	329	45.9 (42.2–49.6)	41.3 (36.1–46.8)	58.7 (53.2–63.9)

## Data Availability

The data used to support the findings of this study are available from the corresponding author upon reasonable request.

## Supplementary Materials

Supplementary Materials Supplementary 1: the relationship between respondents being hypertensive and various risk factors.
