# Supplementary material for: Prevalence of Hypertension in the Middle Belt of Ghana: A Community-Based Screening Study
Source: Int J Hypertens. 2019 Oct 7;2019:1089578. doi: 10.1155/2019/1089578 (PMC6800906; doi:10.1155/2019/1089578)
Supplement: Supplementary Materials — Supplementary 1: the relationship between respondents being hypertensive and various risk factors. [file 1089578.f1.pdf]

Supplementary 1: The relationship between respondents being hypertensive and various risk factors.

	Respondent's Age group			P – Value
**Factor**	**18 – 39 years N=150**	**40 – 59 years N=309**	**≥60 years N=258**	
	n (%)	n (%)	n (%)	
	**Being hypertensive and tobacco use**			
**Current smokers**	0 (0.0)	10 (3.2)	4 (1.6)	**0.053**
**Past tobacco users**	5 (3.3)	22 (7.1)	34 (13.2)	**0.001**
**Any smokeless tobacco users**	3 (2.0)	2 (0.6)	1 (0.4)	**0.201**
	**Being hypertensive and alcohol use**			
	**18 – 39 years N=150**	**40 – 59 years N=309**	**≥60 years N=258**	**P – Value**
**Ever Consumed Alcohol**	50 (33.3)	118 (38.2)	102 (39.5)	**0.445**
**Consumed Alcohol in the past 12 months**	33 (22.0)	66 (21.4)	56 (21.7)	**0.987**
**Consumed Alcohol in the past 30 Days**	28 (18.7)	57 (18.4)	48 (18.6)	**0.998**
	**Being hypertension and other factors**			
	**18 – 39 years N=150**	**40 – 59 years N=309**	**60 & Above years N=258**	**P – Value**
**Vigorous work**	141 (94.0)	288 (93.2)	180 (69.8)	**< 0.001**
**Active in Sports**	52 (34.7)	68 (22.0)	34 (13.2)	**<0.001**
**Diabetic**	8 (5.3)	34 (11.0)	27 (10.5)	**0.131**
**Pre – Diabetes**	9 (6.0)	37 (12.0)	18 (7.0)	**0.042**
**BMI**				
**Underweight**	9 (6.0)	27 (8.7)	50 (19.4)	
**Normal**	84 (56.0)	166 (53.7)	150 (58.1)	**<0.001**
**Overweight**	40 (26.7)	69 (22.3)	32 (12.4)	

**Obese**	17 (11.3)	47 (15.2)	26 (10.1)	
**Fasting Blood Glucose**				
**Normal**	83 (55.3)	131 (42.4)	117 (45.3)	
**Hypoglycemia**	4 (2.7)	11 (3.6)	3 (1.2)	**0.038**
**Hyperglycemia**	63 (42.0)	167 (54.0)	138 (53.5)	
**Blood Cholesterol**				
**Normal**	82 (55.0)	132 (43.8)	92 (37.2)	
**Hypocholesterolemia**	3 (2.0)	4 (1.3)	7 (2.8)	**0.010**
**Hypercholesterolemia**	64 (42.9)	165 (54.8)	148 (59.9)	
**Triglycerides in Blood**				
**Normal**	82 (55.8)	146 (48.5)	112 (44.4)	
**Hypotriglyceridemia**	1 (0.7)	1 (0.3)	1 (0.4)	**0.267**
**Hypertriglyceridemia**	64 (43.5)	154 (51.2)	139 (55.2)	
**Proteinuria (g/L)**				
**Negative**	94 (63.1)	219 (71.1)	164 (63.8)	
**Trace**	49 (32.9)	83 (26.9)	80 (31.1)	
**0.3**	4 (2.7)	2 (0.6)	7 (2.7)	**0.088**
**1.0**	2 (1.3)	0 (0.0)	4 (1.6)	
**3.0**	0 (0.0)	0 (0.0)	1 (0.4)	
**Greater or equal 20**	0 (0.0)	4 (1.3)	1 (0.4)	
